# Chemogenetic Stimulation and Silencing of the Insula, Amygdala, Nucleus Accumbens, and Their Connections Differentially Modulate Alcohol Drinking in Rats

**DOI:** 10.3389/fnbeh.2020.580849

**Published:** 2020-11-04

**Authors:** Mia Haaranen, Annika Schäfer, Vilja Järvi, Petri Hyytiä

**Affiliations:** Department of Pharmacology, Faculty of Medicine, Medicum, University of Helsinki, Helsinki, Finland

**Keywords:** alcohol drinking, DREADDs, insula, amygdala, nucleus accumbens, neural circuits

## Abstract

The anterior insular cortex is hypothesized to represent interoceptive effects of drug reward in the service of goal-directed behavior. The insula is richly connected, but the insula circuitry in addiction remains poorly characterized. We examined the involvement of the anterior insula, amygdala, and nucleus accumbens, as well as the projections of the anterior insula to the central amygdala, basolateral amygdala (BLA), and nucleus accumbens core in voluntary alcohol drinking. We trained alcohol-preferring Alko Alcohol (AA) rats to drink alcohol during intermittent 2-h sessions. We then expressed excitatory or inhibitory designer receptors [designer receptors exclusively activated by designer drugs (DREADDs)] in the anterior insula, nucleus accumbens, or amygdala by means of adenovirus-mediated gene transfer and activated the DREADDs with clozapine-N-oxide (CNO) prior to the drinking sessions. Next, to examine the role of specific insula projections, we expressed FLEX-DREADDs in the efferent insula → nucleus accumbens core, insula → central amygdala, and insula → BLA projections by means of a retrograde AAV-Cre vector injected into the insula projection areas. In the anterior insula and amygdala, excitatory Gq-DREADDs significantly attenuated alcohol consumption. In contrast, in the nucleus accumbens, the Gq-DREADD stimulation increased alcohol drinking, and the inhibitory Gi-DREADDs suppressed it. The Gq-DREADDs expressed in the insula → nucleus accumbens core and insula → central amygdala projections increased alcohol intake, whereas inhibition of these projections had no effect. These data demonstrate that the anterior insula, along with the amygdala and nucleus accumbens, has a key role in controlling alcohol drinking by providing excitatory input to the central amygdala and nucleus accumbens to enhance alcohol reward.

## Introduction

Corticolimbic regulatory systems have long been thought to be the primary neural substrates in addictive behaviors. There is increasing evidence that also the insular cortex (insula) plays an important role in controlling drug taking and seeking. The insula is hypothesized to represent the interoceptive effects of drug taking and integrate them with emotional and decision-making processes (Naqvi and Bechara, 2009; Droutman et al., 2015b). Lesions of insula suppressed smoking-related urges in smokers (Naqvi et al., 2007) and decreased both the conditioned drug effects and drug self-administration in various animal models of addiction (Contreras et al., 2007; Scott and Hiroi, 2011; Cosme et al., 2015; Pushparaj and Le Foll, 2015; Pushparaj et al., 2015). Drug-associated cues activated the insula, suggesting sensitized insula functions during drug craving (Claus et al., 2011; Engelmann et al., 2012). On the other hand, human addicts also displayed lower insula activation while performing decision-making and risk evaluation tasks (Li et al., 2009; Stewart et al., 2014). The apparently discordant findings show that insula may exhibit either sensitized or desensitized functions depending on the behavioral context and possibly the stage of addiction.

The role of insula in mediating drug-related responses can only be understood by studying the networks in which insula is embedded. Insula is connected extensively to the cortical and subcortical brain regions that serve sensory, emotional, and motivational functions (Droutman et al., 2015a). Goal-directed behavior is associated particularly with the anterior agranular insula that differs from the more posterior granular insula by the disappearance of the cortical layer IV (Bermudez-Rattoni, 2014). The anterior agranular insula has reciprocal connections with the anterior cingulate cortex, ventromedial prefrontal cortex, amygdala, and striatal areas, including nucleus accumbens (Shi and Cassell, 1998; Reynolds and Zahm, 2005). Activation of the anterior insula connections with the nucleus accumbens and amygdala is now emerging as an important neural correlate of drug seeking and craving. In humans, increased coupling between the anterior insula and nucleus accumbens was correlated with self-reported compulsive alcohol use (Grodin et al., 2018), and optogenetic inhibition of this projection suppressed alcohol self-administration associated with adverse effects of alcohol in rats (Seif et al., 2013). Similarly, the anterior insula influences drug seeking through connections with the amygdala, shown by increased insula–amygdala connectivity in heroin and cocaine users (Gu et al., 2010; Xie et al., 2011). In rats, relapse to methamphetamine seeking increased c-Fos expression in the central amygdala, whereas chemogenetic inhibition of the anterior insula projection to the central amygdala decreased methamphetamine seeking (Venniro et al., 2017).

Information on the role of the anterior insula connections in mediating drug intake and seeking is still scanty. We designed the present study to clarify further the role of insula and its downstream connections in the regulation of voluntary alcohol drinking during limited alcohol access that models aspects of binge drinking. We used the alcohol-preferring Alko Alcohol (AA) rats that were among the first rat lines developed for high voluntary alcohol drinking using directional selection and represent therefore an enriched genetic contribution to excessive alcohol use (Eriksson, 1968; Sommer et al., 2006). During limited access, these rats display pharmacologically relevant blood alcohol levels that induce psychomotor stimulatory effects (Päivärinta and Korpi, 1993).

Our first goal was to examine the effects of bidirectional manipulation of both the anterior insula, nucleus accumbens, and amygdala separately on alcohol drinking. Although these brain areas, particularly the nucleus accumbens and amygdala, have well-known roles in alcohol reward (Koob et al., 1998; Gilpin et al., 2015), we decided to use the possibility to both stimulate and silence them using designer receptors exclusively activated by designer drugs (DREADDs) that tap into the intracellular signaling cascades and alter neuronal excitability upon administration of clozapine-N-oxide (CNO; Urban and Roth, 2015). Our second goal was to examine the role of the anterior insula projections to the nucleus accumbens core and amygdala subdivisions. We accomplished this by using Cre-dependent DREADDs that were expressed specifically in these projections with the help of a powerful retrograde Cre-carrying adenoviral vector (Tervo et al., 2016) injected into the nucleus accumbens core, central amygdala, and basolateral amygdala (BLA). We hypothesized that bidirectional control of the anterior insula, amygdala, and nucleus accumbens excitability, added with the insula projection-specific manipulation, would give novel information on the role of these brain areas and their connectivity in mediating alcohol reward and drinking and promote ideas of circuit-based treatments for alcohol use disorders.

## Materials and Methods

### Animals

One hundred and seventy-nine male AA rats, bred at the University of Helsinki (Helsinki, Finland) and approximately 10 weeks old at the start of experiments, were used for this study. Rats were maintained at the animal facility of the University of Helsinki in a temperature (20 ± 1°C) and humidity-controlled (55 ± 10%) room with a 12-h light/12-h dark cycle (lights on at 6:00) and housed in individually ventilated cages (IVCs) with access to food (SDS, Witham, UK) and water *ad libitum*. Cages were enriched with nest and bedding material, a wooden block, and a PVC tube. Experiments were authorized by the project authorization board of the Regional State Administrative Agency for Southern Finland (license number ESAVI/1172/04.10.07/2018) and followed Directive 2010/63/EU of the European Parliament and of the Council of the European Union on the protection of animals used for scientific purposes and Finnish Act 497/2013 on the Protection of Animals Used for Scientific or Educational Purposes.

### Intermittent Alcohol Drinking

**Figure 1:**
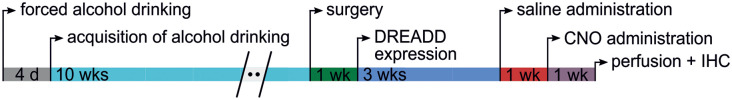
Timeline of the experiments. Shown are the experimental phases and their approximate length each subject was exposed to during the experiments.

To accustom rats to the taste of ethanol, rats first underwent 4 days of forced ethanol drinking. During these days, rats had access to a 10% (v/v) ethanol solution (WWR International, Fontaney-sur-Bois, France) from two 350-ml bottles equipped with stainless steel spouts but no access to water. After these 4 days, an intermittent drinking paradigm commenced. For 2 h, three times weekly (Monday, Wednesday, and Friday from approximately 10:00–12:00), rats had access to water and a 10% ethanol solution in a two-bottle choice paradigm. The fluids were offered in custom-made pipettes with stainless steel spouts that allowed measurement of fluid intake to the nearest 0.1 ml. The side of the cage with the ethanol pipette (left or right) was alternated each drinking session to control for a possible side preference for ethanol consumption. During training, ethanol drinking levels gradually increased and reached an asymptotic level in approximately 8–10 weeks. Rats were weighed weekly for health assessment and calculation of ethanol consumption per kilogram of body weight. Before surgical procedures, each animal batch was divided into three experimental groups [one for the excitatory Gq-DREADD vector, one for the inhibitory Gi-DREADD vector, and one for the control enhanced green fluorescent protein (EGFP) vector] matched for the mean alcohol intake and its variance during the last week. The general timeline for the experiments is depicted in Figure 1.

### Surgery

Animals were anesthetized for 4 min in 5% isoflurane (Vetflurane 1,000 mg/g; VIRBAC S.A., Carros, France) in oxygen (flowrate, 0.8–1 l/min), after which they were fixed onto a stereotaxic frame (David Kopf Instruments, Tujunga, CA, USA) with the incisor bar set to 3.3 mm below the interaural line. Isoflurane anesthesia was continued initially at 3.5% and then at 2.5% isoflurane in oxygen. A drop of Viscotears (Novartis Healthcare A/S, Copenhagen, Denmark) was applied on eyes to prevent them from drying out. Holes were drilled onto the skull, and viral vector injections were performed using a 5-μl syringe (Hamilton 65460-02 Neuros Syringe with a 33-gauge needle, Hamilton Company, Reno, NV, USA) mounted on a stereotaxic injector (Stoelting no. 53313, Wood Dale, IL, USA). The coordinates (mm) for the bilateral injections were as follows: anterior insula, anterior posterior (AP) +3.0 from Bregma, mediolateral (ML) ± 4.2 from the sagittal suture, and ventrodorsal (VD) −6.1 from the skull surface; nucleus accumbens, AP +1.9, ML ± 1.5, VD −7.3; nucleus accumbens core, AP +1.9, ML ± 1.7, VD −7.3; amygdala, AP −2.4, ML ± 4.6, VD −8.2; central nucleus of amygdala, AP −2.2, ML ± 4.6, VD −8.2; BLA, AP −2.2, ML ± 5.1, DV −8.7. All coordinates were based on the Paxinos and Watson (2007).

The following vectors were used for single-site DREADD and EGFP expression: the excitatory Gq-DREADD vector ssAAV-8/2-hSyn1-hM3D(Gq)-mCherry-WPRE-hGHp(A) [5.4 × 10^12^ vector genomes (vg)/ml], the inhibitory Gi-DREADD vector ssAAV-8/2-hSyn1-hM4D(Gi)-mCherry-WPRE-hGHp(A) (7.3 × 10^12^ vg/ml), and the EGFP control vector ssAAV-8/2-hSyn1-EGFP-hGHp(A) (9.4 × 10^12^ vg/ml). For the projection-specific expression of DREADDs, the following vectors were used: the excitatory Gq-DREADD vector ssAAV-8/2-hSyn1-dlox-hM3D(Gq)-mCherry(rev)-dlox-WPRE-hGHp(A) (4.0 × 10^12^ vg/ml), the inhibitory Gi-DREADD vector ssAAV-8/2-hSyn1-dlox-hM4D(Gi)-mCherry(rev)-dlox-WPRE-hGHp(A) (7.4 × 10^12^ vg/ml), and the EGFP control vector ssAAV-8/2-hSyn1-dlox-eGFP(rev)-dlox-WPRE-hGHp(A) (8.0 × 10^12^ vg/ml). The retrograde vector for Cre-recombinase expression was ssAAV-retro/2-hSyn1-chl-EGFP_2A_iCre-WPRE-SV40p(A) (8.0 × 10^12^ vg/ml). Finally, for tracing the anterior insula projections into the subdivisions of the amygdala and nucleus accumbens, we used the following vectors: ssAAV-retro/2-hSyn1-chl-EGFP_2A_iCre-WPRE-SV40p(A) and ssAAV-retro/2-hSyn1-chl-mCherry-WPRE-SV40p(A). The atlas coordinates for the central amygdala were AP −2.2, ML ± 4.6, VD −8.2, and for the BLA, AP −2.2, ML ± 5.1, VD −8.7. The coordinates for the nucleus accumbens core were AP +1.9, ML ± 1.7, VD −7.3, and for the shell, AP +1.9, ML ± 0.8, VD −7.8. All viral vectors were produced by the Viral Vector Facility (VVF) of the Neuroscience Center Zürich (Zentrum für Neurowissenschaften Zürich, ZNZ).

Viral vectors were injected over 3 min, with a 3-min diffusion time. The injection volume for the single-site injections was 0.75 μl, and for injections targeting specific projections, 0.6 μl. In the tracing experiments, the volume of 0.5 μl was used. Carprofen (5 mg/kg s.c., Norbrook Laboratories, Newry, UK) was administered for postoperative analgesia. After surgery, animals were returned to their home cages, and drinking sessions were resumed after two suspended sessions.

### Drugs

DREADD expression was allowed to accumulate for 4 weeks before systemic saline and CNO (ab141704, Abcam, Cambridge, UK) injections. Rats were first injected i.p. with saline 60 min prior to a drinking session to habituate them to the injection procedure and to verify that the injection procedure did not cause any adverse reactions impacting alcohol drinking. After two additional sessions, rats were injected i.p. with CNO (10 mg/kg, dissolved in saline) to activate DREADDs 60 min prior to a drinking session. Ethanol and water intake during the sessions were measured. We chose the 10 mg/kg CNO dose to maximally activate both Gq- and Gi-DREADDs without disturbing motivated behaviors or locomotion, as reported previously (Farrell and Roth, 2013; Mahler and Aston-Jones, 2018; Haaranen et al., 2020). We also controlled the possible nonspecific off-target effects by including an EGFP-expressing non-DREADD group in all experiments. Each subject received CNO only once.

### Immunohistochemistry

After completion of the experiments, animals were deeply anesthetized first with 5% isoflurane and then with a 120 mg/kg lethal dose of pentobarbital (Mebunat vet 60 mg/ml; Orion Pharma, Espoo, Finland). Rats were perfused transcardially with ice-cold phosphate-buffered saline (PBS, +4°C, pH 7.4) followed by 4% paraformaldehyde (PFA, +4°C, pH 7.4). The brains were removed and placed in PFA for a 24-h postfixation, after which PFA was replaced with 30% sucrose in PBS until brains were saturated (~4 days). Brains frozen in isopentane were stored at −80°C until cutting with a freezing microtome into 40-μm coronal sections that were cryoprotected at −20°C.

DREADD expression at the injected brain areas was visualized by immunohistochemical detection of mCherry tagged to DREADDs. Brain sections were washed three times in PBS for 5 min and blocked at room temperature (RT) for 1 h in a blocking solution containing 3% bovine serum albumin (BSA), 10% donkey serum, and 0.3% Triton solubilized in PBS. Sections were incubated overnight at +4°C with the 1:800 rabbit anti-mCherry primary antibody (ab167453, Abcam, Cambridge, UK), followed by a 2-h incubation with the 1:1,000 secondary donkey antirabbit antibody (ab150076, Abcam) at RT. Sections were then mounted on microscopic slides, coverslipped with Vectashield-DAPI (Vectashield + DAPI, Vector Laboratories Inc., Burlingame, CA, USA), and imaged with a Zeiss AxioImager.Z1 upright epifluorescent microscope using the ZEN Blue software. Animals with bilateral DREADD expression at the intended brain areas were included in the data analysis. Six rats (3.4% of all subjects) were discarded due to failure to detect DREADD expression.

### Statistical Analysis

We determined the sample sizes of the experiments based on similar published behavioral studies in rats. In all drinking experiments, the data were expressed as baseline alcohol intake (g/kg) compared with the intake on the CNO injection day. The baseline was determined as the mean of the saline injection day and the following baseline day preceding the CNO injection. The differences between the groups expressing EGFP, Gq-DREADDs, or Gi-DREADDs were analyzed with two-way (session, DREADD vector) repeated measures ANOVA, with repeated measures on session. Differences between the baseline and CNO days were compared with paired *t*-tests. The level for statistical significance was set at *p* < 0.05.

## Results

**Figure 2:**
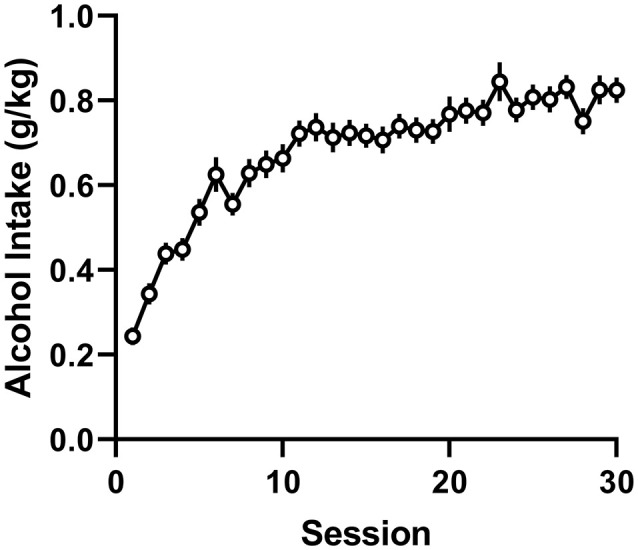
Acquisition of alcohol drinking during intermittent 2-h sessions by alcohol-preferring Alko Alcohol (AA) rats. The data are depicted as mean (±SEM) alcohol intake (g/kg body weight in 2 h) over 30 sessions (10 weeks) by all animals included in the final data analysis (*n* = 173).

**Figure 3:**
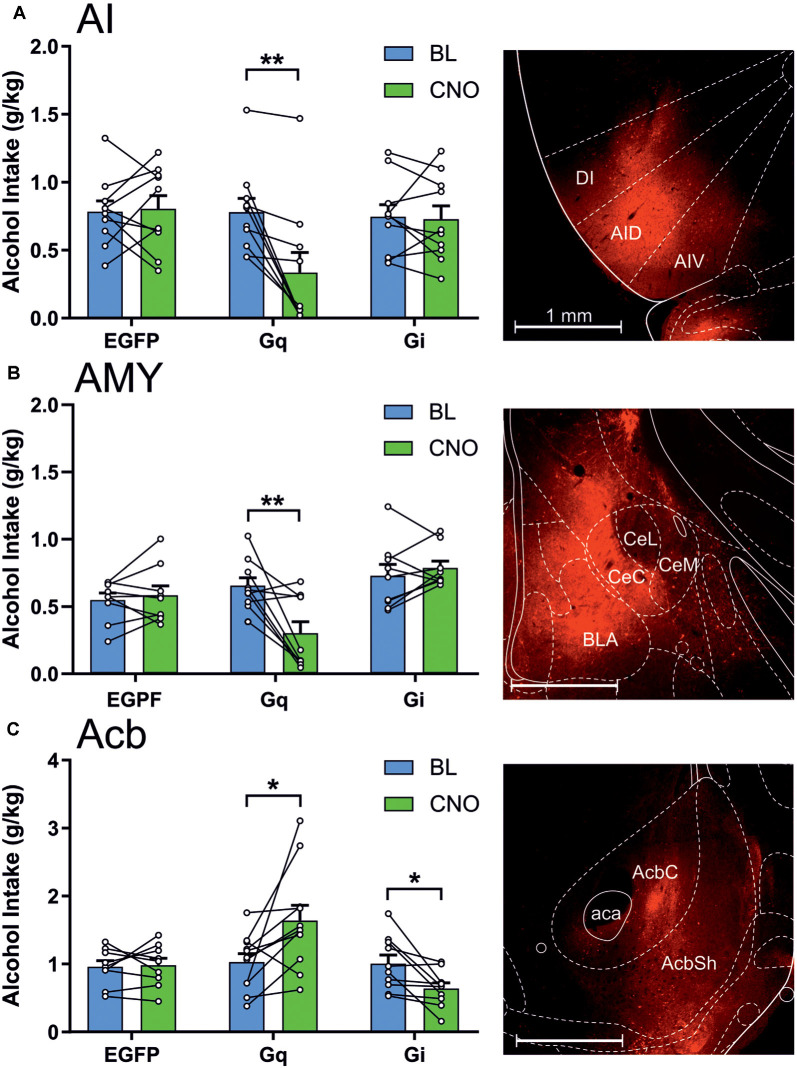
Chemogenetic stimulation and inhibition have differential effects on alcohol drinking in the anterior insula, amygdala, and nucleus accumbens. The data are expressed as mean ± SEM alcohol intake (g/kg) during 2-h sessions during the baseline (BL) and following a clozapine-N-oxide (CNO) injection in three separate treatment groups expressing enhanced green fluorescent protein (EGFP), Gq-designer receptors exclusively activated by designer drugs (Gq-DREADDs), or Gi-DREADDs in each brain area. **(A)** EGFP (*n* = 10), Gq-DREADDs (*n* = 10), and G-DREADDs (*n* = 10) expressed in the anterior insula. **(B)** EGFP (*n* = 9), Gq-DREADDs (*n* = 10), and G-DREADDs (*n* = 9) expressed in the amygdala. **(C)** EGFP (*n* = 9), Gq-DREADDs (*n* = 11), and G-DREADDs (*n* = 10) expressed in the nucleus accumbens. Each histogram is accompanied by a representative brain section expressing Gq-DREADD-tagged mCherry reporter. *p < 0.05, **p < 0.01, paired *t*-tests comparing the BL and CNO sessions. Abbreviations used: DI, dysgranular anterior insula; AID, agranular anterior insula, dorsal; AIV, agranular anterior insula, ventral; CeC, central amygdaloid nucleus, capsular division; CeL, central amygdaloid nucleus, lateral division; CeM, central amygdaloid nucleus, medial division; BLA, basolateral amygdala; AcbC, nucleus accumbens core; AcbSh, nucleus accumbens shell; aca, anterior commissure.

During the intermittent 2-h sessions given three times per week for 10 weeks, AA rats included in the final data analysis exhibited a significant increase in alcohol drinking, as shown by a significant effect for session (*F*_(29,4,988)_ = 31.56, *p* < 0.0001; Figure 2). The mean intake of all included subjects was 0.80 ± 0.02 g/kg during the 10th acquisition week. In these AA rats, we first tested the involvement of the anterior insula, nucleus accumbens, and amygdala in alcohol drinking by expressing the stimulatory Gq- and inhibitory Gi-DREADDs, as well as the EGPF protein as a control in these brain areas using adenovirus-mediated gene transfer (Figure 3). As shown by the representative coronal sections, in the insula, the expression of Gq-DREADDs displayed by mCherry immunoreactivity was largely confined in the anterior agranular insula, encompassing both the ventral and dorsal subdivisions (Figure 3A). Supplementary Figure 1 depicting the maximal and minimal mCherry expression at three Bregma levels for all experimental groups shows mCherry expression also in the granular subdivision in the more posterior insula regions. In the amygdala, DREADD expression was seen both in the central amygdala and to some degree also in the basolateral and basomedial amygdala (Figure 3B and Supplementary Figure 1), and injections into the nucleus accumbens produced DREADD expression both in the core and shell accumbal compartments (Figure 3C and Supplementary Figure 1). When we challenged these animals by giving them CNO 60 min before the onset of the alcohol drinking sessions, we saw significant changes in alcohol consumption in animals expressing the DREADDs in all three brains areas. First, as a replication of our previously published data (Haaranen et al., 2020), CNO significantly altered alcohol intake in rats expressing Gq-DREADDs in the insula, as shown by a significant effect for session (*F*_(1,27)_ = 7.31, *p* = 0.012) and a significant session × vector interaction (*F*_(2,27)_ = 7.60, *p* = 0.002). A comparison of the CNO challenge sessions with the baseline sessions indicated that the 54% decrease in drinking produced by the Gq-DREADDs was significant (*t*_(9)_ = 4.00, *p* = 0.003), with no changes in the other CNO-challenged groups. In a similar fashion, we saw a significant effect of vector (*F*_(2,25)_ = 6.52, *p* = 0.005) and a session × vector interaction (*F*_(2,25)_ = 9.45, *p* = 0.001) in rats expressing Gq-DREADDs in the amygdala. Also in these rats, the only significant change was the 60% decrease in alcohol intake produced by the Gq-DREADD stimulation (*t*_(9)_ = 3.56, *p* = 0.006). Both in the insula and amygdala, the effects of Gq-DREADD activation were bimodal, i.e., three rats in the insula experiment and four rats in the amygdala experiment exhibited no effects or a minor decrease in alcohol drinking. However, when we inspected the extent of mCherry expression in these subjects, they did not differ from subjects with the largest suppression, suggesting that the behavioral effects produced by Gq-DREADD activation were not correlated with DREADD expression. Chemogenetic manipulation altered alcohol drinking also in rats expressing DREADDs in the nucleus accumbens, as revealed by a significant effect of vector (*F*_(2,27)_ = 5.88, *p* = 0.008) and a significant session × vector interaction (*F*_(2,27)_ = 8.83, *p* = 0.001). In contrast to the insula and amygdala manipulation, in the nucleus accumbens, the effects were bidirectional. We found both a significant 59% increase in alcohol drinking by the stimulatory Gq-DREADDs (*t*_(10)_ = 2.67, *p* = 0.024) and a 36% decrease by the inhibitory Gi-DREADDs (*t*_(9)_ = 2.99, *p* = 0.015), indicating that the failure to see effects by Gi-DREADD-mediated neuronal inhibition in the insula and amygdala was not due to the Gi-DREADD vector used.

We also recorded the volume of water offered concomitantly with alcohol during the drinking sessions. Generally, the rats consumed only small amounts of water, from 1 to 3 ml during 2 h, and therefore, any decreases in water consumption would be difficult to record. We observed no significant changes in water intake, indicated by the lack of significant session × vector interactions (insula, *F*_(2,27)_ = 0.40, *p* = 0.67; amygdala, *F*_(2,25)_ = 1.67, *p* = 0.21; nucleus accumbens, *F*_(2,27)_ = 2.26, *p* = 0.12).

**Figure 4:**
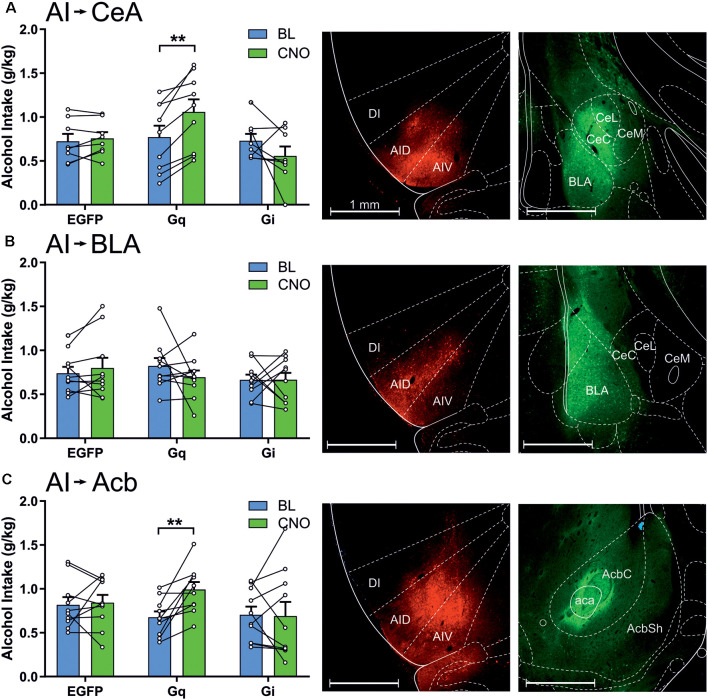
Chemogenetic stimulation of the anterior insula efferents to the central amygdala and nucleus accumbens core increases alcohol drinking. The data are expressed as mean ± SEM alcohol intake (g/kg) during 2-h sessions during the BL and following a CNO injection in three separate treatment groups expressing EGFP, Gq-DREADDs, or Gi-DREADDs in each insula projection. **(A)** EGFP (*n* = 8), Gq-DREADDs (*n* = 9), and G-DREADDs (*n* = 8) expressed in the anterior insula efferents to the central amygdala. **(B)** EGFP (*n* = 10), Gq-DREADDs (*n* = 10), and G-DREADDs (*n* = 10) expressed in the insula efferents to the BLA. **(C)** EGFP (*n* = 10), Gq-DREADDs (*n* = 10), and G-DREADDs (*n* = 10) expressed in the insula efferents to the nucleus accumbens core. Each histogram is accompanied by representative brain sections expressing Gq-DREADD-tagged mCherry reporter in the anterior insula and the AAV2-retro-Cre-tagged GPF in the central amygdala, BLA, and nucleus accumbens core. **p < 0.01, paired *t*-tests comparing the BL and CNO sessions. Abbreviations used: DI, dysgranular anterior insula; AID, agranular anterior insula, dorsal; AIV, agranular anterior insula, ventral; CeC, central amygdaloid nucleus, capsular division; CeL, central amygdaloid nucleus, lateral division; CeM, central amygdaloid nucleus, medial division; BLA, basolateral amygdala; AcbC, nucleus accumbens core; AcbSh, nucleus accumbens shell; aca, anterior commissure.

We next investigated how the efferent projections of the anterior insula to the amygdala and nucleus accumbens regulate alcohol consumption. Projection-specific DREADD expression was accomplished by injecting the Cre-dependent DREADD vectors into the anterior insula and injecting a powerful retrograde AAV-retro-Cre vector either to the central nucleus of the amygdala, BLA, or nucleus accumbens core (Figure 4 and Supplementary Figure 2). When we challenged the animals expressing DREADDs in the projections to the amygdala, only the projection to the central amygdala was involved in alcohol drinking, shown by a significant session × vector interaction (*F*_(2,22)_ = 8.47, *p* = 0.002), whereas the projection to the BLA had no role (session × vector interaction, *F*_(2,27)_ = 1.01, *p* = 0.38). A more detailed analysis showed that, in the central amygdala projection, the Gq-DREADDs produced a significant 27% increase in alcohol drinking (*t*_(8)_ = 5.08, *p* = 0.001), whereas the inhibition of this projection had no effect. In addition, the insula projection to the nucleus accumbens core affected alcohol consumption, shown by a significant session × vector interaction (*F*_(2,27)_ = 3.36, *p* = 0.050). Similar to the central amygdala projection, only the Gq-DREADDs altered alcohol drinking by producing a 47% increase (*t*_(9)_ = 3.70, *p* = 0.005). Projection-specific manipulation produced no significant effects on water consumption (session × vector interactions, insula to central amygdala, *F*_(2,22)_ = 0.00, *p* = 0.99; insula to BLA, *F*_(2,27)_ = 1.47, *p* = 0.25; insula to nucleus accumbens core, *F*_(2,27)_ = 0.07, *p* = 0.93).

**Figure 5:**
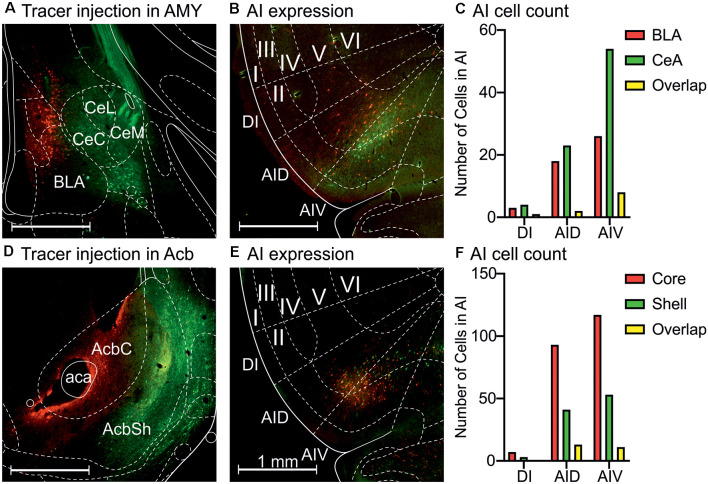
Retrograde tracing with AAV2-retro vectors shows populations of anterior insula neurons projecting to the amygdala and nucleus accumbens subdivisions. **(A)** Starter neurons in the central amygdala (GFP) and BLA (mCherry). **(B)** Retrogradely labeled neurons in the agranular insula. **(C)** Quantification of the DI, AID, and AIV neurons projecting to central (CeA) and basolateral (BLA) amygdala. **(D)** Starter neurons in the nucleus accumbens core (mCherry) and shell (GFP). **(E)** Retrogradely labeled neurons in the agranular insula. **(F)** Quantification of the DI, AID, and AIV neurons projecting to nucleus accumbens core and shell. Abbreviations used: DI, dysgranular anterior insula; AID, agranular anterior insula, dorsal; AIV, agranular anterior insula, ventral; CeC, central amygdaloid nucleus, capsular division; CeL, central amygdaloid nucleus, lateral division; CeM, central amygdaloid nucleus, medial division; BLA, basolateral amygdala; AcbC, nucleus accumbens core; AcbSh, nucleus accumbens shell; aca, anterior commissure. The Roman numerals refer to cortical layers I–VI.

Projection-specific DREADD expression suggested that the anterior insula neurons sending projections to the central amygdala and BLA, as well as the nucleus accumbens, consist of overlapping neuronal populations. To visualize these populations in more detail, we injected either mCherry- or GFP-expressing AAV2-retro vectors into the central or basolateral amygdaloid nuclei, as well as the core and shell accumbal compartments (Figures 5A–F). The central amygdala-projecting neurons comprised 58% of the labeled amygdala-projecting neurons, whereas 34% of neurons projected to the BLA (Figure 5C). The amygdala-projecting neuros exhibited equal numbers in the dorsal agranular insula, but the neurons projecting to the central amygdala were more numerous in the ventral insula. The anterior insula sections showed that the nucleus accumbens core- and shell-projecting anterior insula neurons exhibited the same distribution pattern in the insula, localized mainly in the dorsal and ventral agranular anterior insula, with very few neurons in the dysgranular insula (Figure 5E). However, quantification of the labeled neurons revealed that the core-projecting neurons were more abundant (64% of neurons) than the shell-projecting neurons (29%; Figure 5F), in accordance with earlier findings (Reynolds and Zahm, 2005). In all cases, projection neuron locations were largely confined to cortical layers II and V.

## Discussion

In the present work, we used both excitatory and inhibitory DREADDs for examining the role of the forebrain circuitry comprised of the anterior insula, amygdala, and nucleus accumbens in voluntary alcohol drinking in alcohol-preferring rats. We found that chemogenetic stimulation of the anterior insula and amygdala robustly suppressed alcohol consumption, whereas inhibition of these brain areas had no effect. In contrast, nucleus accumbens stimulation increased alcohol drinking, and the inhibition of this area decreased it. Projection-specific chemogenic manipulation of the insula efferents showed that the excitatory insula inputs to the central amygdala and nucleus accumbens core stimulated alcohol drinking, whereas inhibition of these efferents failed to affect it. Together, these data show the importance of both the anterior insula, amygdala, and nucleus accumbens in the regulation of alcohol drinking and suggest that the excitatory input from the anterior insula to the central amygdala and nucleus accumbens core enhances the reinforcing properties of alcohol. We have previously shown that chemogenetic stimulation of the anterior insula also suppressed sucrose consumption (Haaranen et al., 2020), warranting further studies to test the effects produced by DREADDs in the nucleus accumbens, amygdala, and the insula projections to these areas on consummatory behaviors in general.

In a replication of our previous study, we found that anterior insula stimulation by the Gq-DREADDs reduced alcohol consumption (Haaranen et al., 2020). The simple limited access alcohol drinking model does not allow us to analyze which aspects of drinking behavior were affected by insula stimulation apart from the consumed alcohol volume. Insula mediates taste processing and the interoceptive effects of alcohol (Maffei et al., 2012; Jaramillo et al., 2016), but it is unlikely that these factors contributed to suppressed drinking because many animals hardly sampled alcohol after insula stimulation. This could suggest that insula stimulation causes aversive states or general malaise. We have recently demonstrated with c-Fos mapping and pharmacological magnetic resonance imaging that anterior insula stimulation activates also the more posterior insula areas (Haaranen et al., 2020). Optogenetic or chemogenetic stimulation of the posterior insula induced freezing and escape movements, and stimulation of posterior insula projections to the central amygdala caused aversive states and avoidance behavior (Schiff et al., 2018; Gehrlach et al., 2019). In our experiments, however, anterior insula stimulation failed to produce freezing or escape reactions, and both locomotor activity and water drinking induced by mild water deprivation remained intact (Haaranen et al., 2020), suggesting that the stimulation effects were related to hedonic processes, in accordance with earlier studies mapping appetitive processes to anterior insula regions (Peng et al., 2015). Indeed, anterior insula electric stimulation given during presentation of liquid reward-associated conditioned stimuli attenuated appetitive approach behavior in primates (Saga et al., 2018).

The failure to see changes in alcohol drinking by insula inhibition by Gi-DREADDs appears inconsistent in the light of previous data showing that insula lesions or inactivation decreased drug consumption and their conditioned effects in various animal models (Scott and Hiroi, 2011; Contreras et al., 2012; Cosme et al., 2015; Pushparaj and Le Foll, 2015; Pushparaj et al., 2015). The fact that the same Gi-DREADDs expressed in the nucleus accumbens decreased alcohol intake in this study shows that these DREADDs were fully functional, suggesting that the efficacy of inhibitory DREADDs to alter behavior may depend on the neuron type or brain area in which they are expressed. In addition, the crucial difference between lesioning or inactivation methods and chemogenetic inhibition is that the latter results only in a partial suppression of neuronal firing (Chang et al., 2015; Smith et al., 2016). There is also evidence that DREADD-induced neuronal stimulation may produce much more robust effects than inhibition (Chang et al., 2015). Finally, one of the possible factors for determining the effects of neuronal inhibition in the anterior insula is the behavioral context of inhibition. For example, insula inhibition may preferentially suppress drug seeking or consumption in contexts associated with adverse consequences (Seif et al., 2013; Campbell et al., 2019).

As our DREADD injections into the amygdala produced DREADD expression in the central as well as the basolateral and basomedial amygdala subdivisions, DREADD-induced behavioral effects cannot be conveniently ascribed to any individual amygdala subdivision. The largely GABAergic central nucleus is divided further in the central lateral (CeL) and central medial (CeM) nuclei. The CeL receives projections from various cortical and subcortical brain areas through the lateral amygdala and projects to the CeM that receives projections also from the BLA, which is a cortical-like structure with glutamatergic projection neurons and GABAergic interneurons (Duvarci and Pare, 2014). The CeM is the main output nucleus of the amygdala, projecting to behavioral and physiological effector regions (Gilpin et al., 2015).

Amygdala chemogenetic stimulation suppressed alcohol drinking in a remarkably similar manner as in the anterior insula, an effect reminiscent of central amygdala ibotenic acid lesions, blockage of GABA_A_ receptors, or ablation of neurotensin neurons (Hyytiä and Koob, 1995; Möller et al., 1997; Torruella-Suarez et al., 2020). All central amygdala subdivisions harbor GABAergic neurons defined by the expression of specific molecular markers. Both the CeM and CeL contain corticotropin-releasing hormone (CRH) neurons. Their chemogenetic activation increased anxiety-like behavior in mice, possibly through CRH projections to the locus coeruleus, periaqueductal gray, parabrachial nucleus, and bed nucleus of stria terminalis (BNST; Paretkar and Dimitrov, 2018), whereas their inactivation decreased escalation of alcohol drinking and somatic withdrawal symptoms induced by dependance, mediated by CRH projections to the BNST (de Guglielmo et al., 2019). In the CeL, on the other hand, optogenetic activation of protein kinase C-δ-expressing neurons attenuated food intake and water drinking (Cai et al., 2014). Similarly, deep brain electrical stimulation (DBS) into the central amygdala stopped rats working for sucrose pellets and consuming them (Ross et al., 2016), in parallel with our findings. Both DBS and chemogenetic stimulation may exert supraphysiological effects, and it could hypothesized that they severely disrupt functions of the intrinsic amygdala circuitry encoding reward and therefore suppress behavior in a manner of lesioning the area (Grill et al., 2004; Ross et al., 2016).

In addition, the BLA has been implicated in reward-related behaviors, often by loss-of-function studies (Wassum and Izquierdo, 2015). The BLA exhibits an intermingled distribution of both positive and negative valence-coding neurons that have different projection targets. For example, neurons projecting to the nucleus accumbens mediate preferentially reward-predicting cues, whereas neurons projecting to the CeM are involved in aversive outcomes (Beyeler et al., 2018). BLA lesions did not affect alcohol drinking (Möller et al., 1997), but optogenetic stimulation of the BLA projection to the nucleus accumbens shell attenuated cued alcohol seeking as well as alcohol drinking (Millan et al., 2017). Recent optogenetic experiments suggested that stimulation of the BLA, while enhancing conditioned approach and appetitive conditioning, does not affect primary reward (Servonnet et al., 2020). In contrast, optogenetic stimulation of a genetically distinct subset of central amygdala neurons was reinforcing (Kim et al., 2017), which is consistent with our data that stimulation of the anterior insula projections into the central amygdala but not the BLA increased alcohol drinking. This finding is in agreement with earlier data that alcohol consumption is positively correlated with neural activation in the central amygdala (Sharko et al., 2013) and that relapse to methamphetamine seeking is associated with an activation of the anterior insula projection to the central amygdala but not to the BLA (Venniro et al., 2017). Finally, these findings suggest a dichotomy of the functional roles of the projections that the central amygdala receives from the anterior and posterior insula. The anterior insula projections appear to promote drug seeking and reward, whereas the posterior insula projections are associated with avoidance behavior and aversive states (Schiff et al., 2018; Gehrlach et al., 2019).

The opposite effects of DREADD-induced stimulation in the anterior insula and the anterior insula projections into the central amygdala appear perplexing, but there could be various reasons for this discrepancy. Because we used the panneuronal human synapsin 1 (hSyn) promotor for expressing the DREADDs, it is possible that Gq-DREADDs were also present in cortical inhibitory interneurons. Their activation could lead to suppression of large ensembles of insula projection neurons. In addition, a general insula stimulation could also target the somas of neurons projecting to other areas than the ones examined in our experiments, and these projections could have inhibitory effects on alcohol drinking. As mentioned above, anterior insula stimulation is also propagated to the posterior insula, and although we do not favor posterior insula-mediated aversion as the explanation for reduced alcohol consumption in our study, we cannot exclude the possibility that interactions between the anterior and posterior insula could produce behavioral effects.

In contrast to the other brain areas examined, chemogenic manipulation of the nucleus accumbens produced bidirectional effects on alcohol drinking. Inhibition of the nucleus accumbens with Gi-DREADDs decreased alcohol drinking, consistent with previous studies with chemogenetic inhibition, transient inactivation, or electrolytic lesions in limited-access alcohol drinking paradigms both in mice and rats (Hodge et al., 1995; Dhaher et al., 2009; Cassataro et al., 2014; Wilden et al., 2014). Stimulation of the nucleus accumbens or the anterior insula projection to the nucleus accumbens core increased alcohol consumption, which replicates the enhancement of reward-related behavior produced by optogenetic stimulation of either the nucleus accumbens or the excitatory accumbal inputs from the ventral hippocampus, prefrontal cortex, or BLA (Britt et al., 2012). Our data therefore suggest that the glutamatergic projection from the anterior insula is one of the various inputs contributing to the total accumbal excitatory input that enhances reward seeking. This finding is also agreement with data from human heavy drinkers showing increased task-dependent connectivity between the anterior insula and nucleus accumbens associated with behavioral compulsivity (Grodin et al., 2018). Given that the insula projection to the nucleus accumbens is only one of the many excitatory inputs increasing accumbal glutamate release, the lack of effect by silencing this projection could be expected. In agreement with this notion, optogenetic inhibition of this projection suppressed only consumption of quinine-adulterated but not unadulterated alcohol (Seif et al., 2013), suggesting that silencing is efficient only for aversion-resistant alcohol consumption. However, chemogenetic inhibition on the insula–accumbens projection reduced alcohol self-administration, when CNO was administered intracranially into the nucleus accumbens (Jaramillo et al., 2018a,b), which may have led to a higher local CNO concentration at Gi-DREADDs than systematically given CNO in our experiments and thereby enhanced neuronal silencing.

To conclude, our present study produced two main outcomes. First, we showed using chemogenetic tools that manipulation of the anterior insula, amygdala, or nucleus accumbens alone is sufficient to alter voluntary alcohol drinking. Second, we provided evidence that excitatory projections from the anterior insula to the nucleus accumbens core and central amygdala enhance alcohol reward. Particularly in the central amygdala with various subdivisions and neuron types identified by molecular markers, the exact circuitry targeted by chemogenetic manipulation remains to be elucidated. All in all, these data suggest that the anterior insula is one of the forebrain hubs regulating alcohol reward through various downstream targets, including the central amygdala and nucleus accumbens.

## Data Availability Statement

The raw data supporting the conclusions of this article will be made available by the authors, without undue reservation.

## Ethics Statement

The animal study was reviewed and approved by Project authorization board of the Regional State Administrative Agency for Southern Finland.

## Author Contributions

PH and MH were responsible for the study concept and design and drafted the manuscript. MH, AS, and VJ contributed to the acquisition of animal data, immunohistochemical analysis, microscopy, and provided critical revision of the manuscript for important intellectual content. All authors contributed to the article and approved the submitted version.

## Conflict of Interest

The authors declare that the research was conducted in the absence of any commercial or financial relationships that could be construed as a potential conflict of interest.
