# Supplementary material for: Chemogenetic Stimulation and Silencing of the Insula, Amygdala, Nucleus Accumbens, and Their Connections Differentially Modulate Alcohol Drinking in Rats
Source: Front Behav Neurosci. 2020 Nov 4;14:580849. doi: 10.3389/fnbeh.2020.580849 (PMC7671963; doi:10.3389/fnbeh.2020.580849)
Supplement: Supplementary file 1 [file Image_1.pdf]

Supplementary Material

Supplementary Figures

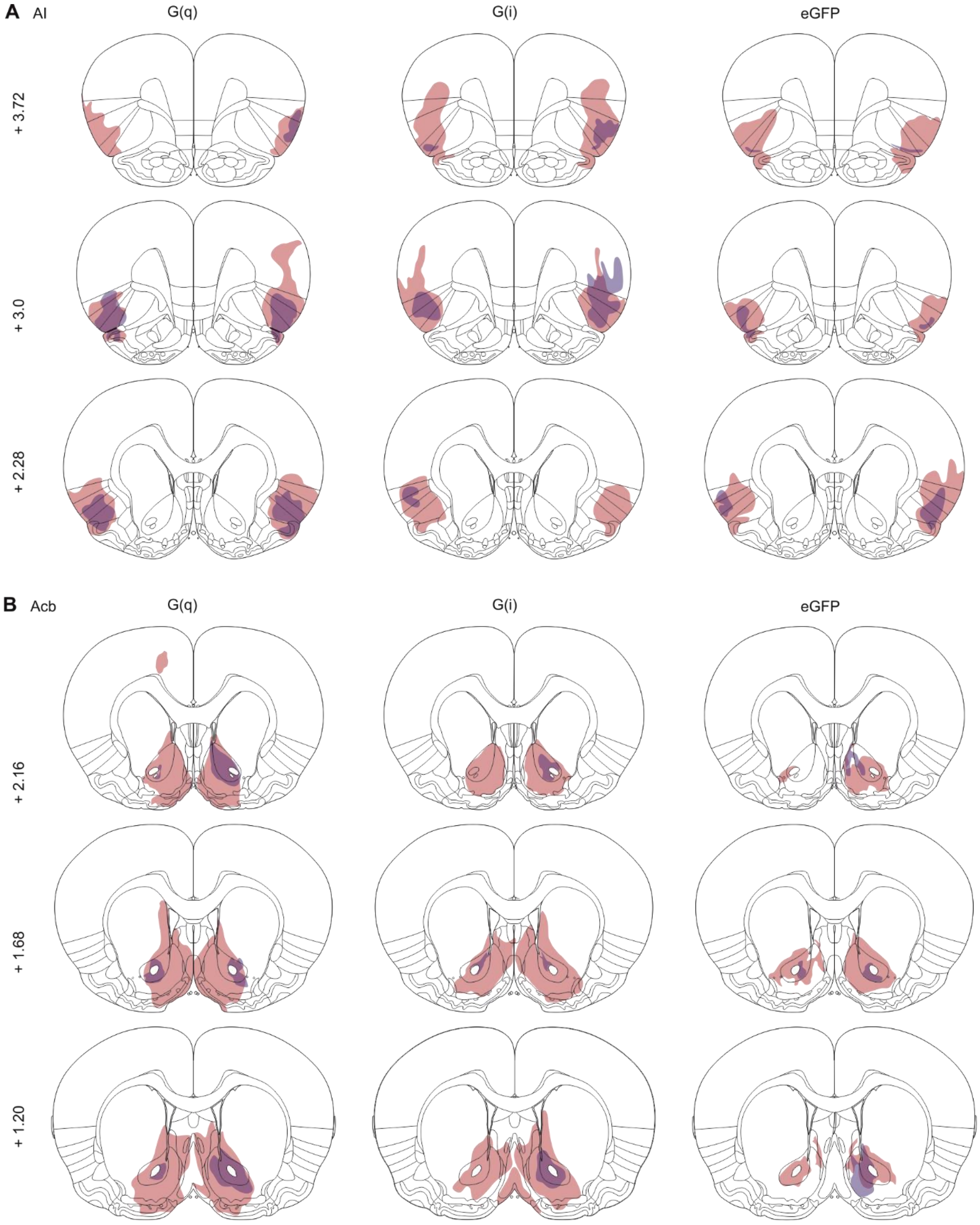

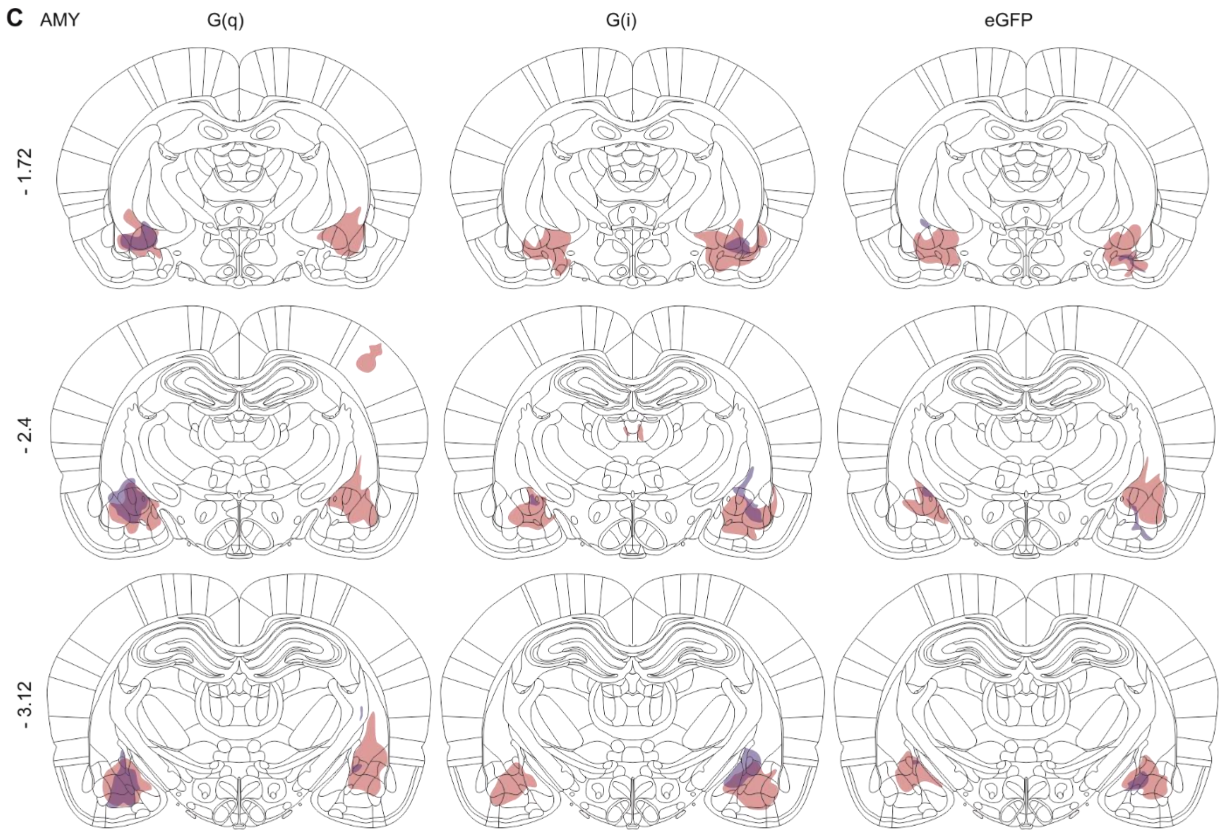

Supplementary Figure 1. Maps of fluorescent reporter expression tagged to DREADD or control vectors at the anterior insula (A), nucleus accumbens (B), and amygdala (C). Left column: mCherry tagged to Gq-DREADDs, middle column: mCherry tagged to Gi-DREADDs, right column: EGFP tagged to non-DREADD control vectors. The middle row for all brain areas shows the intended injection sites and the rows above and below the more anterior and posterior sections. The bregma coordinates are based on the Paxinos and Watson rat brain atlas, 6th edition, 2007. The maximum expression is depicted as magenta and the minimum as blue.

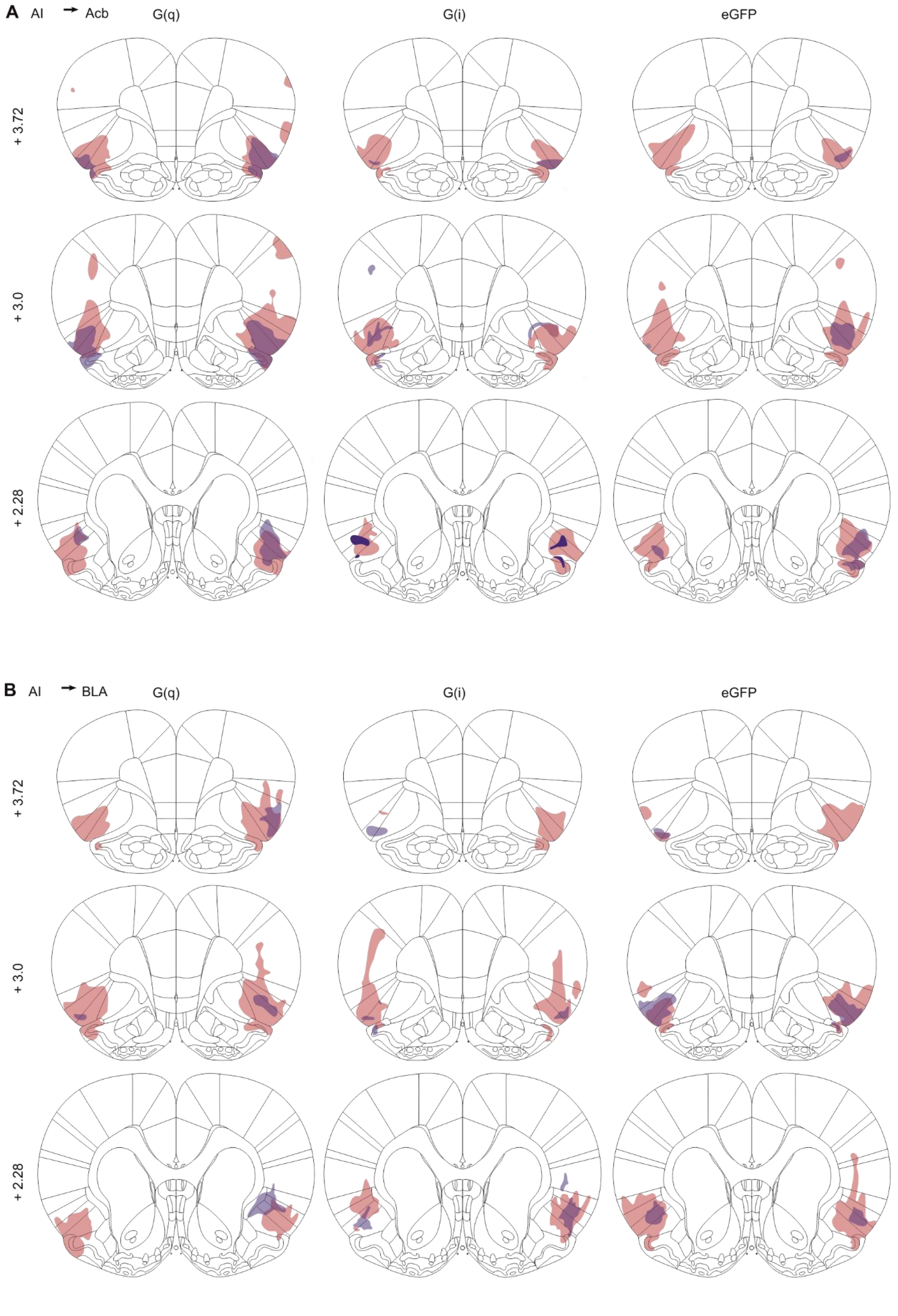

A AI → Acb
G(q)
G(i)
eGFP
+ 3.72
+ 3.0
+ 2.28
B AI → BLA
G(q)
G(i)
eGFP
+ 3.72
+ 3.0
+ 2.28

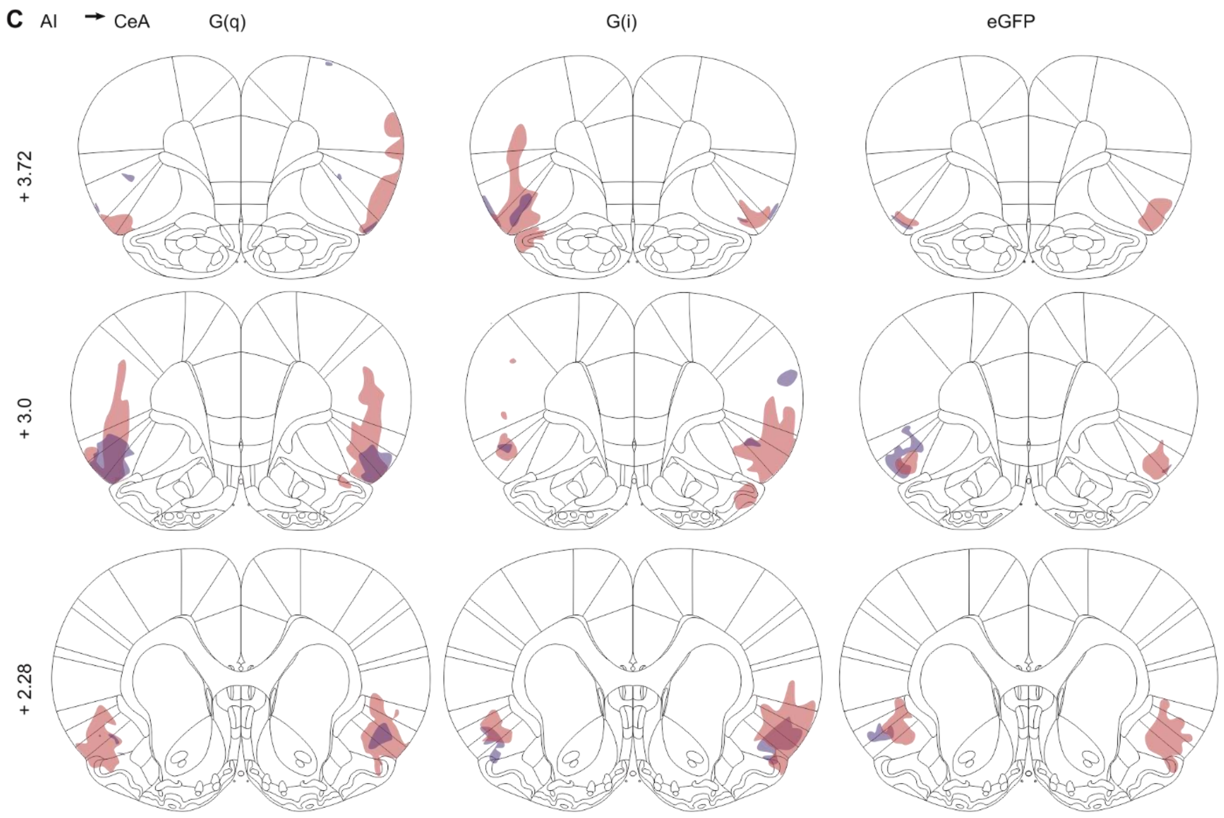

Supplementary Figure 2. Maps of fluorescent reporter expression tagged to FLEX-DREADDs or control vectors at the anterior insula following AAV2-retro-Cre injections into the nucleus accumbens core (A), basolateral amygdala (B), or central amygdala (C). Left: mCherry tagged to Gq-DREADDs, middle: mCherry tagged to Gi-DREADDs, right: EGFP tagged to non-DREADD control vectors. The middle row shows the intended injection site for the FLEX-DREADD injections at the anterior insula and the rows above and below the more anterior and posterior sections. The bregma coordinates are based on the Paxinos and Watson rat brain atlas, 6th edition, 2007. The maximum expression is depicted as magenta, and the minimum as blue.
